# Supplementary material for: Influenza A Virus Infection Reactivates Human Endogenous Retroviruses Associated with Modulation of Antiviral Immunity
Source: Viruses. 2022 Jul 21;14(7):1591. doi: 10.3390/v14071591 (PMC9320126; doi:10.3390/v14071591)
Supplement: Supplementary file 1 [file viruses-14-01591-s001.zip › MotifAnalysis.zip!/MotifOutput_SC35M/homerResults/motif33.similar.html]

motif33

## Information for motif33

C
G
T
A
C
G
T
A
A
C
G
T
A
C
T
G
A
C
G
T
C
G
T
A
A
G
T
C
A
C
G
T
A
C
G
T
A
C
G
T
A
C
T
G
A
C
G
T
  
Reverse Opposite:  

C
G
T
A
A
G
T
C
C
G
T
A
C
G
T
A
C
G
T
A
A
C
T
G
C
G
A
T
C
G
T
A
A
G
T
C
G
T
C
A
A
C
G
T
A
C
G
T
  

|  |  |
| --- | --- |
| p-value: | 1e-4 |
| log p-value: | -9.539e+00 |
| Information Content per bp: | 1.938 |
| Number of Target Sequences with motif | 2.0 |
| Percentage of Target Sequences with motif | 4.88% |
| Number of Background Sequences with motif | 14.8 |
| Percentage of Background Sequences with motif | 0.03% |
| Average Position of motif in Targets | 81.8 +/- 39.5bp |
| Average Position of motif in Background | 142.4 +/- 40.1bp |
| Strand Bias (log2 ratio + to - strand density) | 10.0 |
| Multiplicity (# of sites on avg that occur together) | 2.00 |
| Motif File: | file (matrix) reverse opposite |

### Similar de novo motifs found

|  |  |  |  |  |  |  |  |
| --- | --- | --- | --- | --- | --- | --- | --- |
| Rank | Match Score | Redundant Motif | P-value | log P-value | % of Targets | % of Background | Motif file |
| 1 | 0.852 | A C T G A C G T C G T A A G T C A C G T A C G T A C G T A C T G A C G T C T G A | 1e-3 | -8.996225 | 7.32% | 0.23% | motif file (matrix) |
| 2 | 0.826 | A C G T C G T A A G T C A C G T A C G T A C G T A C T G A C G T | 1e-2 | -5.223092 | 7.32% | 0.86% | motif file (matrix) |
